# Fistulating diverticular disease masquerading as a peri-anal abscess: a laparoscopic approach to management

**DOI:** 10.1093/jscr/rjab483

**Published:** 2021-11-05

**Authors:** Emily Schmidt, Matthew Corbitt, Krish Kulendran, Boris Ruggiero

**Affiliations:** Surgical Department, Cairns Hospital, Cairns, Queensland, Australia; Surgical Department, Cairns Hospital, Cairns, Queensland, Australia; Surgical Department, Cairns Hospital, Cairns, Queensland, Australia; Surgical Department, Cairns Hospital, Cairns, Queensland, Australia

## Abstract

We present a rare case of complicated sigmoid diverticulitis presenting as a peri-anal abscess from an extra-sphincteric fistulous tract. This presentation of a colocutaneous peri-anal abscess is extremely rare, with only a handful of cases described in the literature. Most are managed with an open sigmoid colectomy, however, this case was successfully managed laparoscopically. It highlights the need to consider extra-levator causes of peri-anal abscess, such as pelvic sepsis causing fistulating disease, and to consider early magnetic resonance imaging if there is clinical suspicion of underlying pathology. It also demonstrates that a safe and potentially less morbid outcome is possible via laparoscopic approach when compared to traditional open surgical approach.

## INTRODUCTION

We present a rare case of complicated sigmoid diverticulitis presenting as a perianal abscess from an extra-sphincteric fistulous tract. Only a handful of cases are described in the literature, which have been mostly managed with open sigmoid colectomy. This case was successfully managed laparoscopically. Usually, presentations of peri-anal abscess proceed swiftly to examination under anesthetic (EUA). This case highlights the need to consider extra-levator causes of peri-anal abscess and to have low threshold for early imaging. It also demonstrates a good outcome through first managing the sepsis, then proceeding with a laparoscopic approach to definitive surgical management once the disease was quiescent.

## CASE REPORT

A 55-year-old male presented with a 1-month history of perianal swelling with increasing discomfort. Other than malaise, he remained systemically well. He reported 3 months of intermittent abdominal discomfort, thought to be related to constipation, which had resolved by the time of his presentation. He had no prior history of perianal abscess and had a medical history that was significant only for conservatively managed diverticulitis, with a recent colonoscopy confirming mild disease. On peri-anal examination, there was fullness and tenderness over the right ischiorectal space. The tenderness extended from 9 to 11 o’clock ~4 cm from the anal verge. No external opening was obvious, and there were no masses on per rectal exam. His abdomen was soft and non-tender.

Bloods upon presentation revealed a mild leucocytosis (white cell count of 12.5 × 10^9^/l). An external computed tomography (CT) scan performed 3 weeks prior showed sigmoid diverticulitis for which the patient had not been treated. A tiny pocket (<1 cm) of extra-luminal air was noted, but there was no abscess or collection. When reviewing these external images, there was suspicion of a right peri-anal abscess.

The patient was commenced on intravenous antibiotics and underwent EUA and drainage of perianal abscess. An ischiorectal cavity was found with small amount of purulent material. No fistulae into the rectum were identified. Further imaging was considered at thistime.

**Figure 1:**
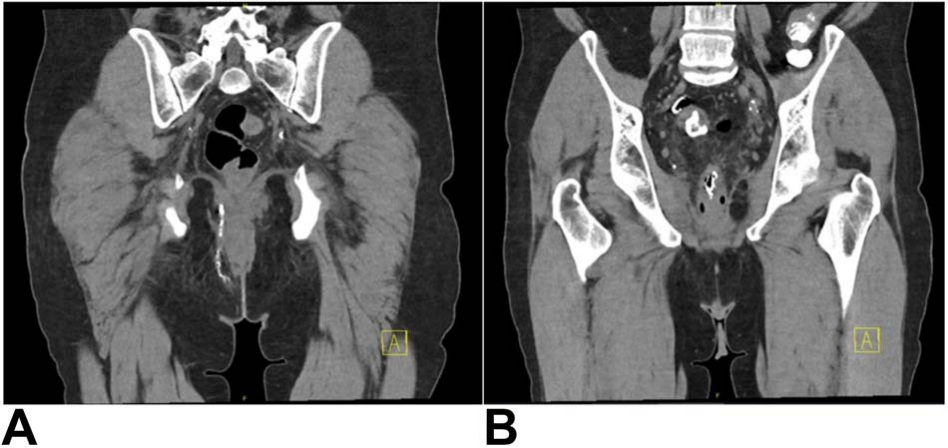
(**A**) CT fistulogram showing a long fistulous tract extending from the right peri-anal region, superiorly in the ischiorectal fossa, traversing the inferior aspect of the levator ani and extending around the right of the anal canal and rectum; (**B**) superiorly, it extended into a thick-walled inflamed segment of sigmoid colon with numerous diverticulae; contrast can be seen entering the sigmoidloop.

**Figure 2:**
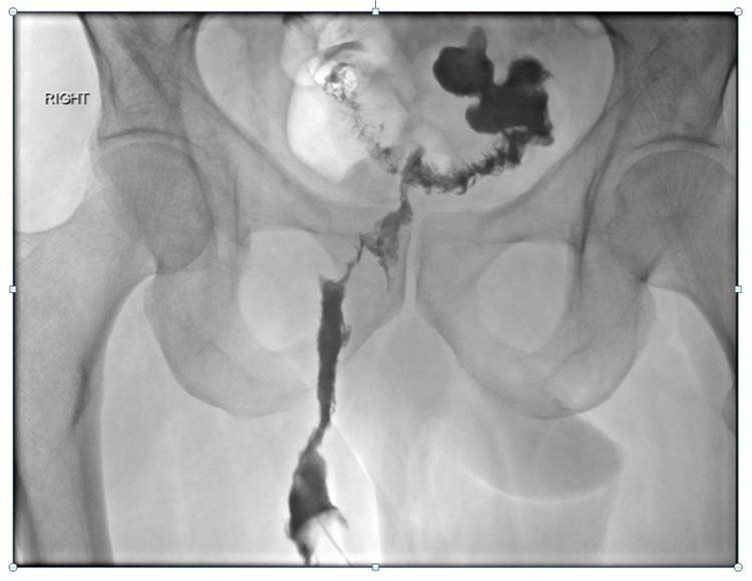
Fistulogram of colocutaneous peri-anal fistula.

Magnetic resonance imaging (MRI) identified a fistulous tract extending from the sigmoid colon, around the anterior aspect of the anal canal, with small collections in the ischiorectal and ischioanal fat. CT fistulogram showed a long fistulous tract extending from the right peri-anal region to a sigmoid diverticulum above the recto-sigmoid junction (Figs 1 and 2). The patient underwent a second EUA at which a large tract was identified at 9 o’clock, running parallel to the anus and rectum, with no apparent connection to the anus but draining enteric content. The superior extent of the tract was not felt. Colonoscopy showed a benign-appearing, intrinsic moderate sigmoid stenosis measuring 2 cm in length, which was traversed with difficulty.

**Figure 3:**
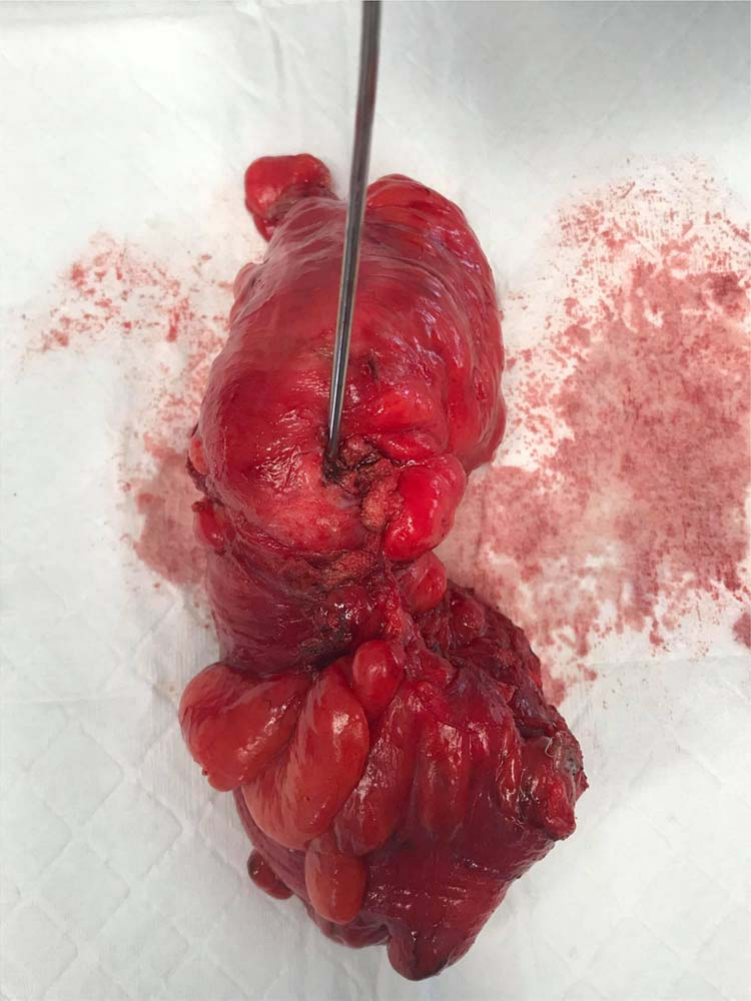
Surgical specimen showing opening of the fistula tract from a sigmoid colon diverticulum

The patient underwent an elective laparoscopic Hartman’s procedure, with prophylactic ureteric stenting. Intra-operatively, significant inflammation at Denovillier’s fascia was found. The recto-sigmoid junction was diseased with chronic diverticulitis and numerous indurated appendages were attached to the pelvic sidewall. The rectum was predominantly healthy. A fistulous opening was laparoscopically visualized at 11 o’clock, originating at the junction of the TME plane (Fig. 3). This opening was dealt with by performing a sigmoid colectomy. The tract through the levator was curetted and managed with placement of a low pelvic drain. Given the proximity of the fistulous opening and inflammation, a decision was made not to re-anastomose immediately and a colostomy was formed.

Histopathology showed benign diverticular disease. The patient recovered well post-operatively and was discharged on Day 5. He has since been followed up in the outpatients’ clinic and is well. He will be considered for reversal of Hartmanns’ following review of MRI and rectal gastrograffin enema to ensure all peri-anal tracts are addressed prior.

## DISCUSSION

Diverticulitis and its complications are common and can include fistulae, predominantly colovesical or colovaginal. Only 1% of diverticular fistulae are colocutaneous in nature [1]. These occur most often on a background of previous interventional drainage of diverticular abscess, or previous bowel resection [2]. Peri-anal colocutaneous fistulae are an even rarer subset, with only isolated cases reported in the literature [1, 3–7]. Anal fistulous disease has traditionally been described using the Parks classification [8]. This case, in addition to emerging cases in the literature, highlights the need to consider beyond the Parks classification to secondary causes of peri-anal disease, such as pelvic sepsis.

Presentation with seemingly acute peri-anal abscess can conceal the underlying cause of fistulating diverticular disease, which can delay diagnosis and management. In our case, lack of abdominal pain at presentation masked the contribution of underlying active diverticulitis. This patient also lacked previous risk factors of colocutaneous fistulating disease [3]. Colocutaneous fistulae are unlikely to heal spontaneously and require operative intervention [3] after initial sepsis control with abscess drainage and antibiotics. Definitive management requires bowel resection. Open procedures are traditionally preferred in these cases due to intra-abdominal adhesions, subsequent difficulties identifying dissection planes and the associated difficulties with abdominal access [1, 3, 5]. An open approach with high anterior resection and defunctioning loop ileosteomy, with excision and packing of the external fistulae opening has been successful in the management of peri-anal fistula from diverticulitis [3]. Ayoubi *et al.* described an uncomplicated post-op course, healing of the external opening and no residual fistula or anastamotic leak on follow-up gastrograffin studies [3]. Two cases in the literature described an attempted laparoscopic approach. However, both required conversion to lower midline laparotomy secondary to difficulties with dissection due to adhesions and scarring [1, 7]. One case underwent anastamosis post-sigmoid resection and was managed with temporary defunctioning loop ileostomy [1], while another had primary colo-rectal anastomosis alone. Peri-anal wounds were both curetted and packed, and both cases had unremarkable recovery periods with healing of the fistulae [1, 7].

There has been previous reported successful laparoscopic management of colocutaneous fistula to the groin [9]. Our case is the first reported case of successful laparoscopic management of colocutnaeous fistula to the peri-anal region, with a laparoscopic Hartman’s procedure and temporizing end-colostomy. Although there was extensive inflammation intra-operatively, dissection was completed laparoscopically and conversion to midline laparotomy was not required. Steps were taken pre-operatively to reduce the risks of the dissection, such as placement of bilateral ureteric stents to assist in ureteric preservation. Due to the extensive inflammation, an anastomosis was not suitable with risk of recurrence of the fistula, and hence, our patient will require a second stage procedure with reversal in the future. Laparoscopic management for diverticulitis has been shown to have shorter hospital stays and decreased overall complication rates, including surgical site infections, prolonged ventilation times and sepsis, compared to open approach [10].

Diverticular disease presenting with peri-anal abscess from spontaneous colocutaneous fistula is rare. This case highlights the importance of considering occult causes of peri-anal abscess and demonstrates successful laparoscopic management of such acase.

## CONFLICT OF INTEREST STATEMENT

None declared.

## FUNDING

None.
